# Liver-derived endocrine IGF-I is not critical for activation of skeletal muscle protein synthesis following oral feeding

**DOI:** 10.1186/1472-6793-13-7

**Published:** 2013-05-08

**Authors:** Britt-Marie Iresjö, Johan Svensson, Claes Ohlsson, Kent Lundholm

**Affiliations:** 1 Department of Surgery Sahlgrenska University Hospital Kir., Metabol lab Bruna Stråket 20-413 45, Gothenburg Sweden; 2 Department of Internal Medicine, Sahlgrenska Academy, University of Gothenburg, Gothenburg, Sweden

**Keywords:** IGF-I, Muscle protein synthesis, Cell signaling, Amino acid

## Abstract

**Background:**

Insulin-like growth factor-1 (IGF-1) is produced in various tissues to stimulate protein synthesis under different conditions. It is however, difficult to distinguish effects by locally produced IGF-1 compared to liver-derived IGF-1 appearing in the circulation. In the present study the role of liver-derived endocrine IGF-I for activation of skeletal muscle protein synthesis following feeding was evaluated.

**Results:**

Transgenic female mice with selective knockout of the IGF-I gene in hepatocytes were freely fed, starved overnight and subsequently refed for 3 hours and compared to wild types (wt). Liver IGF-I knockout mice had 70% reduced plasma IGF-I. Starvation decreased and refeeding increased muscle protein synthesis (p < 0.01), similarly in both IGF-I knockouts and wt mice. Phosphorylation of p70s6k and mTOR increased and 4EBP1 bound to eIF4E decreased in both IGF-I knockouts and wt mice after refeeding (p < 0.05). Muscle transcripts of IGF-I decreased and IGF-I receptor increased (p < 0.01) in wild types during starvation but similar alterations did not reach significance in knockouts (p>0.05). mTOR mRNA increased in knockouts only during starvation. Plasma glucose decreased during starvation in all groups in parallel to insulin, while plasma IGF-I and GH did not change significantly among the groups during starvation-refeeding. Plasma amino acids declined and increased during starvation-refeeding in wild type mice (p < 0.05), but less so in IGF-I ^(−/−)^ knockouts (p < 0.08).

**Conclusion:**

This study demonstrates that re-synthesis of muscle proteins following starvation is not critically dependent on endocrine liver-derived IGF-I.

## Background

Protein synthesis is rapidly increased in skeletal muscles after oral feeding due to intracellular signaling for activation of initiation or protein translation [1], which is quantitatively the most important alteration in the control of short term protein balance in skeletal muscles [2,3]. Meal feeding is a complex stimuli where nutrients are provided and appear simultaneously with changes in circulating levels of hormones and substrates as well as hormone binding proteins. Our earlier studies have suggested that amino acids are important substrate factor(s) behind post-feeding activation of muscle protein synthesis, whereas insulin seems rather permissive [4,5]. On the other hand, it can be expected that insulin-like growth factor I (IGF-I) should be involved since provision of anti IGF-I antibodies before meal feeding attenuated a subsequent rise in protein synthesis [5], besides its well-recognized effects on muscle cell proliferation and regeneration [6-8]. Furthermore, blood levels and muscle expression of IGF-I are significantly changed during starvation and feeding [5,9]. The role of endocrine liver-derived and locally tissue produced IGF-I to promote protein synthesis in response to feeding is however unclear, in part due to complex conditions with different IGF-binding proteins, which may also have independent control functions of net protein metabolism at tissue levels [10-12]. This complexity makes it uncertain to define roles of locally produced versus endocrine liver-derived IGF-I for activation of protein translation and synthesis in skeletal muscle tissue following feeding [13,14]. The aim of the present study was therefore to evaluate the role of endocrine liver-derived IGF-I for activation of translation initiation of muscle proteins at refeeding in a transgenic mouse model with selectively and conditionally deleted liver IGF-I [15]. This deletion caused levels of circulating IGF-I to be reduced by 70% compared to wild type controls in combination with normal muscle expression of IGF-I [15].

## Methods

### Animals

Transgenic female mice (C57BL/6) with inducible inactivation of the Insulin-like growth factor-1 gene constructed with the Cre/LoxP system were used. Animals were bred as described earlier [15]. Recombination was induced by polyinosinic-polycytidylic acid (PiPc) treatment at 10–12 weeks of age in mice homozygous for loxP and heterozygous for Mx-Cre, [15,16]. These mice are referred to as liver IGF-I knockouts, Li-IGF-I ^(−/−)^. PiPc treated siblings, homozygous for LoxP but lacking Mx-Cre, were used as controls and referred to as wild type animals, WT ^(+/+)^. Adult weight stable Li-IGF-I ^(−/−)^ mice housed with 5 animals per cage, matured normally with normal body composition as compared to our previous experiments on partially IGF-I knockouts ^(+/−)^[9]. Body weight was 22.2±0.1 g in knockouts and 23.8±0.10 g in wild types at the start of experiments, in 5 months old mice; which is before any alterations in body composition in knockouts [17]. Male animals were not used since they must be housed individually in this kind of experiment; a condition which is associated with stress reactions and adaptations. However, females are subjected to hormone alterations during the estrous cycle, which could theoretically induce alterations in muscle protein metabolism. However, comparisons of rates of muscle protein synthesis between phases of the menstrual cycle are limited. Miller *et*.*al*. found no effect of the menstrual cycle phases in human females [18], while Toth *et*.*al*. reported minor changes in the gastrocnemius fractional synthesis rate related to estradiol or progesterone replacement therapy in ovariectomized rats [19]. Therefore, all our experiments were carried out with mice randomly divided across cages housed simultaneously and close together, in order to promote estrous synchronization within and across cages.

Liver IGF-I knockouts and wild type mice were used across the groups; freely fed, starved and refed animals. Freely fed animals had continuous access to water ad lib. and standard rodent chow (2016 Global Tekland®, Netherlands). Starved mice had no access to food overnight for 12 hours before experiments, while refed animals were similarly starved overnight for 12 hours and had then free access to food during 3 hours before termination according to previous findings [4,9,20]. Animals were killed by cervical dislocation and blood samples were drawn by cardiac puncture. Mixed hind limb muscles from both legs were excised. Muscles from one leg were used for measurements of fractional protein synthesis and protein measurements. Muscle tissue from the other leg was used for RNA extraction and RNA expression analyses.

All animal procedures were performed in accordance to Swedish law (Animal welfare act, 1988:538) and national guidelines for care and use of research animals (DFS 2004:4) and approved by the regional ethics committee for animal research in Gothenburg (450–2008).

### RNA expression

Hind limb mixed muscle tissue were snap-frozen in liquid nitrogen and kept in −70°C until analysis. RNA was extracted using RNeasy fibrous tissue kit (Qiagen) with DNAse step included. Total RNA concentrations were measured by spectrophotometer (Nanodrop ND-100) and RNA quality was checked by calculating the 18S/28S ratio using an Agilent 2100 bioanalyser. One μg of total RNA was reverse transcribed using oligo d(T)-primer according to kit instructions (Advantage RT for PCR kit,) for cDNA synthesis. Positive and negative controls were included in each run of cDNA synthesis. Realtime PCR was performed with a LightCycler 1.5 instrument (Roche) using the LightCycler FastStart DNA Master^PLUS^SYBR Green 1 kit. For analysis of Igf-1, 2 μl cDNA and 10 pmol of forward (GCT CTT CAG TTC GTG TGT GGA C) and reverse (CAT CTC CAG TCT CCT CAG ATC) primers were used to each reaction of 20 μl. PCR were performed with the following settings; denaturation 95°C for 10 sec., annealing 64°C, 4 sec. and extension 72°C, 6 sec. For analysis of Igf-1r (Quantitect Primer assay Nr QT00155351, Qiagen), 2 μl cDNA and 2 μl of premixed Quantitect primers were used for each reaction of 20 μl. PCR were performed with the following settings; denaturation 95°C for 10 sec., annealing 60°C, 6 sec. and extension 72°C, 5 sec. For analysis of Akt (QT00114401), mTOR (QT01532916) and PI3-kinase(QT00149709), 2 μl cDNA and 2 μl of premixed Quantitect primers were used for each reaction of 20 μl. Real time PCR were performed using QuantiFast™SYBR Green PCR kit (Qiagen). Quantitative results were produced by the relative standard curve method. All samples were analyzed in duplicates and negative controls were included in each run. All results are related to the expression of Gapdh as housekeeping gene according to separate evaluations among 12 gene alternatives, where Gapdh showed minimal alterations in response to starvation/refeeding with 8% variation among all the groups at extreme conditions as starvation/refeeding (0.15<p>0.80).

### Protein synthesis

Fractional liver and skeletal muscle protein synthesis were measured by the flooding dose technique as described [21,22]. A single dose injection of L-[U-^14^C] phenylalanine (0.4 μCi/g) in 150 mM phenylalanine was provided ip. 30 minutes before killing. Liver tissue and mixed hind limb muscles were rapidly excised and frozen in liquid nitrogen until analysis. Blood samples were heparinized and immediately centrifuged at +4°C and plasma was frozen until analysis. Fractional synthesis rate (% × hr^-1^) was calculated as described [9].

### Western blotting

Mixed hind limb muscles were rapidly excised and frozen in liquid nitrogen until next day when biopsies were thawed and homogenized in seven volumes of ice-cold buffer A (20 mM Hepes, pH 7.4, 100 mM KCl, 0.2 mM EDTA, 2 mM EGTA, 1 mM DTT, 50 mM NaF, 50 mM β−glycerophosphate, 0.1 mM AEBSF, 1 mM bensamidine, 0.5 mM sodium vanadate). The homogenate was centrifuged at 10 000 × g for 10 min at 4°C. Aliquots of the supernatant were used for western blot analysis of phosphorylation state of 4EBP1 and p70s6 kinase or immunoprecipitated for analysis of 4EBP1 · eIF4E complexes as previously described [23]. Anti-eIF4E antibodies used were a kind gift from Dr Scot Kimball, Pennsylvania State University, USA. All blot membranes were exposed to Hyperfilm ECL (Amersham Biosciences, UK) and quantification was carried out with Quantity One software (Bio-Rad Laboratories AB, Sundbyberg, Sweden). For quantification of 4EBP1 · eIF4E complex the total optical density was measured and the results were corrected for eIF4E content. Optical density is expressed in arbitrary units. The phosphorylation state of 4EBP1 is expressed as percentage of the most phosphorylated γ-form compared to the total and calculated as optical density of the γ-band/optical density of all bands [(α+β+γ)*100]. Phosphorylation state of p70s6 kinase was measured by gel mobility shift and is expressed as percentage of the least phosphorylated α form compared to the total and calculated as optical density of the α-band/optical density of all bands [(α+β+γ)*100]. For measurement of mTOR and phosphorylated mTOR^2448^, aliquots of the homogenate supernatant were mixed with equal volumes of 2x SDS electrophoresis sample buffer and separated in a 3-8% NuPage Tris-acetate mini gel (Invitrogen). Proteins were transferred to PVDF membranes, which were incubated over night at +4°C with a rabbit anti mouse mTOR^2448^ antibody (#2971, Cell Signaling Technology) after blocking in 10% nonfat dry milk in Tris-buffered Saline-Tween 20. The blots were then washed, incubated with secondary antibodies and developed using an ECL Western Blotting Kit according to the manufacturers description (Amersham Biosciences, UK) and exposed against Hyperfilm ECL (Amersham Biosciences, UK). After detection of signals, antibodies were removed by 45 minutes incubation at +50°C in stripping buffer (100 mM 2-mercaptoethanol, 2% sodium dodecyl sulfat (SDS), 62.5 mM Tris–HCl pH 7.6) and membranes were thereafter reprobed for measurement of total mTOR by incubating membranes 75 min at room temperature with a rabbit anti human mTOR antibody (sc-8319, Santa Cruz biotechnology, Santa Cruz, USA) Quantification of signals was carried out with GS-710 imaging densitometer and Quantity One software (Bio-Rad Laboratories AB, Sundbyberg, Sweden). Measured optical density was expressed as arbitrary units. On each gel, 2 lanes were loaded with MagicMark XP Western Protein Standards (Invitrogen). The average optical density for the standard band with most similar molecular size as the measured protein was used to normalize signal intensity between blots.

### Western ligand blotting

IGF-I ligand blotting was performed under conditions described by Hosssenlopp et.al. [24]. Equal volumes from each supernatant with similar amount of proteins of the prepared muscle homogenates in one group were pooled and mixed with an equal volume of Laemmli sample buffer and heated to 95°C. 15 μl of the samples were separated by electrophoresis on 12% Tris-Glycine gels using non-reduced conditions and transferred to PVDF membranes. Membranes were blocked and incubated overnight with ^125^I-labelled IGF-I (PerkinElmer). After washing, blots were exposed to Hyperfilm MP at −70°C. Films were scanned and band intensity was measured (Quantity One software, GS-710 densitometer, BioRad). The molecular weight of IGFBPs was estimated from prestained standards.

### Plasma concentrations

Plasma IGF-I was measured by an IGF-I binding protein blocked RIA (Mediagnost). Glucose was measured with the glucose oxidase method according to the manufacturers’ instructions (Roche). Plasma amino acids were analyzed by HPLC as described elsewhere [25,26], in order to relate alterations in Igf-I/Igf-Ir transcript expression to changes in plasma amino acids, which may be stimulators of muscle protein synthesis [20,27]. Growth hormone (GH) and insulin were measured with ELISA methods (Linco research). Plasma IGFBPs were not measured since IGFBPs were estimated in the muscle tissue compartment.

### Statistics

Results are presented as mean ± SEM based on 8 animals in each group except for 7 mice in starved wild type and 9 in Li-IGF-I ^(−/−)^. Factorial ANOVA was used to test within groups and among groups followed by Fisher PLSD test for multiple comparisons. P<0.05 was considered statistically significant and p < 0.10 a trend to significance in two tailed tests.

## Results

### Plasma concentrations

Plasma levels of IGF-I were reduced by 70% in liver IGF-I knockout mice (Li-IGF-I ^(−/−)^) compared to wild type mice at all nutritional conditions (<0.001), while glucose and insulin levels were comparable among Li-IGF-I ^(−/−)^ and wild type mice. Overnight starvation caused a significant decrease in plasma glucose compared to freely fed mice in both knockout and wt mice (p<0.05) (Table 1). Growth hormone did not change significantly within groups during freely feeding, starvation and refeeding and it was not significantly altered when comparing between groups. Branched chain and essential amino acids were significantly altered during starvation-refeeding in wild type mice, but not in liver IGF-I knockouts. These alterations were also reflected in concentrations of the sum of all amino acids (Table 2).

**Table 1:** Plasma concentrations in study (starved, refed) and control mice (freely fed) from wild type and liver IGF-I knockout mice

		**Freely fed**	**Starved**	**Refed**	**Within groups p<**	**Among groups p<**
Glucose (mmol/l)	wt	10.4 ± 0.5	8.0 ± 0.6	11.3 ± 0.4	0.05	
	IGF-	12.3 ± 1.2	8.1 ± 0.6	10.0 ± 0.8	0.05	ns
Insulin (μg/l)	wt	0.58±0.09	0.35±0.05	0.93±0.16	0.01	
	IGF-	1.11±0.26	0.43±0.07	0.98±0.20	0.05	ns
IGF-I (μg/l)	wt	172 ± 13	138 ± 13	159 ± 8	ns	
	IGF-	58 ± 7	40 ± 7	46 ± 7	ns	0.01
GH (μg/l)^a^	wt	2.0±0.8	5.1±2.1	12.6±7.3	ns	
	IGF-	11.5±8.5	15±6.1	17.6±11.3	ns	ns

Mean±SEM.

A measured at 9 a.m.

**Table 2:** Plasma amino acids in freely fed, starved and refed mice as described in material and methods

			**Freely fed**	**Starved**	**Refed**	**Within groups p<0.05**	**Among groups p<0.05**
			μmol/liter				
*Essential AA*							
	Isoleucine	wt	61 ± 6	56 ± 4	67 ± 9	ns	
		IGF-	59 ± 6	72 ± 7	50 ± 6	ns	ns
	Leucine	wt	124 ± 17	84 ± 6	179 ± 29	0.05	
		IGF-	111 ± 12	114 ± 11	131 ± 20	ns	ns
	Valine	wt	161 ± 16	120 ± 6	176 ± 20	ns	
		IGF-	152 ± 12	156 ± 13	143 ± 17	ns	ns
	**BCAA**	**wt**	**346** ± **38**	**260** ± **16**	**422** ± **58**	**0**.**05**	
		**IGF**-	**321** ± **28**	**342** ± **31**	**325** ± **43**	**ns**	ns
	Lysine	wt	258 ± 16	246 ± 16	277 ± 14	ns	
		IGF-	281 ± 21	282 ± 26	273 ± 21	ns	ns
	Methionine	wt	55 ± 7	41 ± 5	97 ± 10	0.001	
		IGF-	48 ± 5	38 ± 3	77 ± 13	0.01	ns
	Phenylalanine	wt	287 ± 37	389 ± 107	386 ± 93	ns	
		IGF-	402 ± 86	277 ± 36	311 ± 72	ns	ns
	Threonine	wt	147 ± 14	119 ± 10	207 ± 13	0.05	
		IGF-	125 ± 7	114 ± 8	162 ± 20	0.05	0.05
	Tryptophan	wt	34 ± 4	30 ± 3	42 ± 4	ns	
		IGF-	36 ± 3	35 ± 4	33 ± 3	ns	ns
	**Essential AA**	**wt**	**1127** ± **80**	**1086** ± **119**	**1433** ± **89**	**0**.**05**	
		**IGF**-	**1213** ± **112**	**1089** ± **70**	**1181** ± **91**	**ns**	ns
*Non*-*essential AA*							
	Alanine	wt	337 ± 33	262 ± 37	547 ± 47	0.001	
		IGF-	283 ± 26	194 ± 13	459 ± 44	0.001	0,01
	Arginine	wt	77 ± 12	20 ± 7	43 ± 10	0.01	
		IGF-	56 ± 14	41 ± 9	30 ± 10	ns	ns
	Aspartic acid	wt	37 ± 6	34 ± 3	25 ± 3	ns	
		IGF-	22 ± 2	40 ± 6	33 ± 4	ns	ns
	Asparagine	wt	31 ± 2	27 ± 2	41 ± 2	0.01	
		IGF-	25 ± 2	25 ± 1	32 ± 3	ns	0.01
	Citrulline	wt	30 ± 3	20 ± 2	34 ± 2	0.01	
		IGF-	29 ± 2	20 ± 1	30 ± 3	0.01	ns
	Glutamic acid	wt	166 ± 18	152 ± 16	129 ± 15	ns	
		IGF-	166 ± 11	138 ± 13	158 ± 23	ns	ns
	Glutamine	wt	538 ± 36	520 ± 30	578 ± 31	ns	
		IGF-	553 ± 27	516 ± 42	560 ± 35	ns	ns
	Glycine	wt	174 ± 18	170 ± 17	178 ± 10	ns	
		IGF-	150 ± 9	140 ± 15	175 ± 24	ns	ns
	Histidine	wt	53 ± 4	50 ± 3	70 ± 4	0.01	
		IGF-	57 ± 4	52 ± 4	64 ± 4	ns	ns
	Serine	wt	108 ± 12	85 ± 5	136 ± 5	0.01	
		IGF-	93 ± 16	75 ± 5	118 ± 9	0.01	ns
	Tyrosine	wt	330 ± 23	395 ± 65	339 ± 82	ns	
		IGF-	323 ± 39	239 ± 40	285 ± 59	ns	ns
	Ornitine	wt	88 ± 22	90 ± 12	109 ± 16	ns	
		IGF-	109 ± 16	83 ± 15	115 ± 10	ns	ns
	α-Aba	wt	7 ± 1	8 ± 1	9 ± 1	ns	
		IGF-	6 ± 1	6 ± 1	8 ± 1	ns	ns
	**Non**-**ess**. **AA**	**wt**	**1977** ± **142**	**1835** ± **149**	**2237** ± **89**	**ns**	
		**IGF**-	**1872** ± **71**	**1570** ± **112**	**2065** ± **125**	**0**.**05**	ns
	**Total AA**	**wt**	**3103** ± **219**	**2921** ± **264**	**3669** ± **143**	**0**.**05**	
		**IGF**-	**3085** ± **171**	**2659** ± **180**	**3246** ± **205**	**0**.**08**	ns

Mean±SEM.

wt = wild type mice.

IGF- = hepatic deficient IGF-I ^(−/−)^ mice.

### Protein synthesis

**Figure 1:**
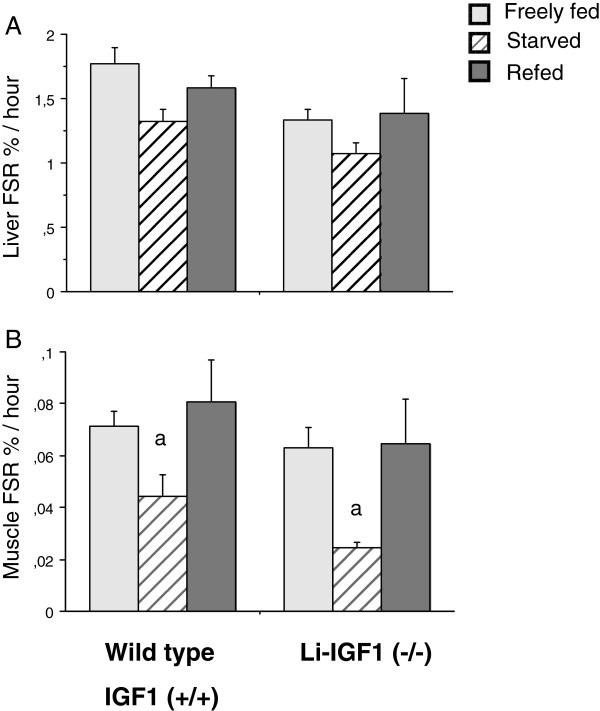
**A,B Effects of starvation-refeeding on synthesis rate of proteins in mixed skeletal muscle and liver tissue.** FSR% was measured with the “flooding dose” technique ([^14^C]-Phe) as described. Animals were freely fed, starved for 12 hours or refed during 3 hours after starvation period as described in Methods. Results are mean ± SEM for 7–9 animals per group. a-p<0.05 vs. freely fed and refed mice.

The magnitude of basal fractional synthesis rate in liver and muscle tissue was comparable in freely fed wild type and liver IGF-I knockout mice. Refeeding increased similarly liver (p < 0.10) and muscle protein synthesis (p < 0.01) compared to starvation in both wild type and liver IGF-I knockout animals (Figure 1A,B).

### RNA transcripts in skeletal muscles

**Figure 2:**
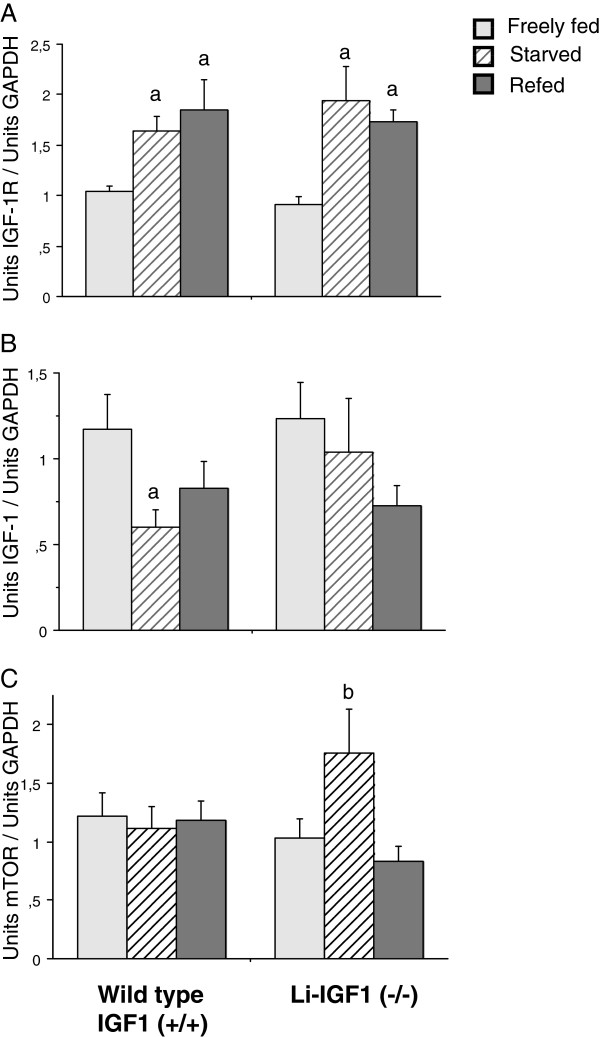
**A-C Effects of starvation-refeeding on Igf-I, Igf-Ir and mTOR mRNA content in skeletal muscle tissue.** Analyzed by quantitative PCR using SYBRgreen detection. Results are mean ± SEM for 7–9 animals per group. a-p<0.05 vs. freely fed animals. b-<0.05 vs. freely fed and refed animals.

mRNA transcript levels were comparable in freely fed wild type and knockout mice (Igf-I, Igf-Ireceptor, PI3-kinase, Akt and mTOR). However, Igf-I transcripts decreased significantly in muscles from wild type mice during starvation but with a different time course in knockouts. Igf-Ir increased similarly during starvation in both wild type and knockout mice and remained increased during refeeding (Figure 2A-B). Significant changes in transcript levels of PI3k and Akt were not observed (PI3-kinase: WT, FF 1.40±0.15, ST 1.12±0.16, RF 1.54±0.21; Li-IGF^(−/−)^, FF 1.13±0.18, ST 1.62±0.17, RF 1.50±0.26) (Akt: WT, FF 1.09±0.15, ST 1.14±0.08, RF 1.41±0.16; Li-IGF^(−/−)^, FF 1.12±0.13, ST 1.54±.24, RF 1.49±0.17). By contrast, mTOR expression was significantly increased in starved knockout animals compared to refed mice without any similar change in wild type mice (Figure 2C). However, this increased transcript level did not correspond to altered mTOR protein levels among animal groups (not shown).

### Translational and cell signaling proteins in skeletal muscle tissue

**Figure 3:**
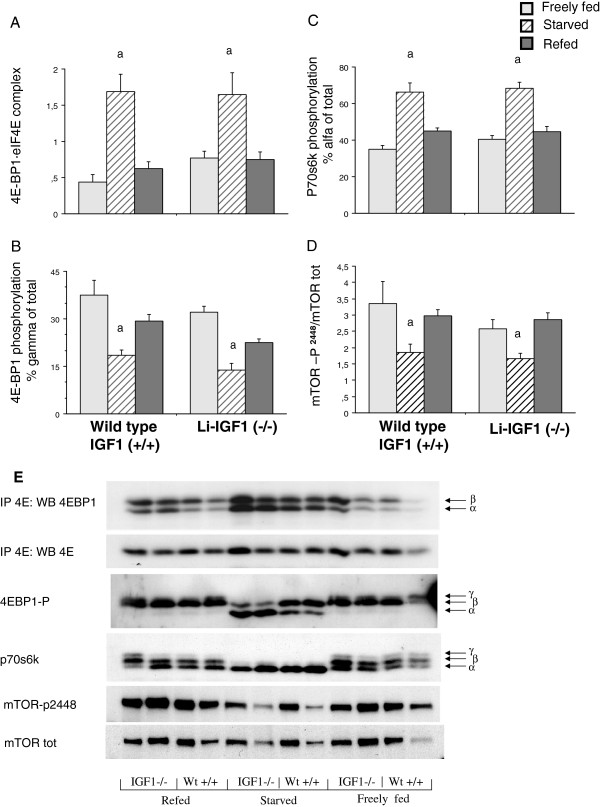
**A**-**E Effects of starvation**-**refeeding on total 4E**-**BP1** **·** **eIF4E complex(A),4E**-**BP1** **·** **phosphorylation(B)p70s6kinase phosphorylation(C)and mTOR phosphorylation in skeletal muscle tissue.** Figure 3E is a representative blot, one of four, of each analysis. Proteins were immunoprecipitated with a monoclonal anti-eIF4E antibody and thereafter analyzed by Western blot with an antibody against 4E-BP1 in analysis of 4E-BP1 · eIF4E complex. Results are mean ± SEM with 7–9 animals per group. a-p<0.01 vs. freely fed and refed mice. All blot analyses were perfomed on the same protein extracts with the same amount of total protein applied among the groups (not shown).

**Figure 4:**
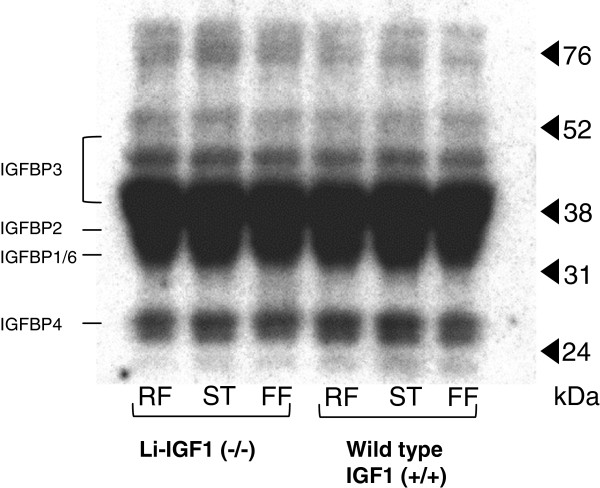
**Radioligand blot which demonstrates similar levels of IGF binding proteins in skeletal muscle tissue among groups in relationship to molecular weights(24**–**76 kD).** Approximate mobility of IGF-I binding proteins 1–6 is indicated to the left. The same amount of proteins (±3%) was loaded in all tracks determined by spectrophotometry.

The 4EBP1 · eIF4E complex, 4EBP1 phosphorylation state (% γ of total), p70s6k phosphorylation (% α of total) and mTOR posporylation (p-mTOR^ser2448^/mTOR^total^) were comparable among wild type and liver IGF-I knockout mice. Starvation significantly increased muscle content of 4EBP1 · eIF4E complexes with corresponding normalization in refed animals (Figure 3A). The 4EBP1 phosphorylation state was significantly and similarly decreased in starved mice compared to freely fed mice with significant increase in refed wild type and liver IGF-I knockout mice (Figure 3B). p70s6k and mTOR^2448^ was less phosphorylated in starved wild type and liver IGF-I knockout mice with complete reversal in both groups during refeeding (Figure 3C,D,E). IGFBP content was similar among all groups (Figure 4) indicating comparable extracellular conditions of binding proteins among the groups.

## Discussion

Anabolic effects by IGF-I are well recognized, particularly such as stimulation of protein synthesis related to cell proliferation, tissue growth and cell differentiation [6,28]. Parts of these effects are probably mediated by muscle tissue specific IGF-I isoforms such as the mechano growth factor [29]. We have earlier assumed that circulating IGF-I is involved in promotion of diurnal alterations of muscle protein synthesis in response to meal feeding [14]; where a similar role may be assumed for insulin [5]. Our earlier studies have, however, not confirmed an important and primary role of insulin in physiologic resynthesis of skeletal muscles during animal starvation-refeeding experiments [4], as well as in human skeletal muscles, where insulin only attenuated break down of non-myofibrillar proteins [2]. Therefore, we assumed that endocrine IGF-I may have a role in stimulation of diurnal variations of protein synthesis in skeletal muscles related to feeding with increased flux of substrates and amino acids across cell membranes [30]. A role of IGF-1 was also tempting, when we observed that locally expressed IGF-I decreased in muscle tissue following overnight starvation and increased in response to meal feeding [5]. However, the complex situation, with protein bound circulating IGF-I and locally produced IGF-I, makes it difficult to demonstrate defined roles for the various IGF-I compartments, particularly when binding proteins themselves may have independent regulatory functions across muscle membranes [11,12]. Therefore, it seemed interesting to apply investigations in liver IGF-I knockout mice, where IGF-I in the circulation is low due to a lack in production of IGF-I in the liver and spleen, in order to re-examine the role of circulating level versus local tissue expression of IGF-I/IGF-IR for alterations in skeletal muscle protein synthesis during starvation-refeeding [5,9].

Earlier attempts to study nutritional effects by IGF-I have not provided unanimous and conclusive results [5,9,31]. We found that injection of anti IGF-I antibodies before feeding of mice attenuated a subsequent rise in protein synthesis by 25%, while ip. injections of IGF-I to overnight starved mice only increased protein synthesis marginally [5], and studies on genetically altered mice implied that fractional resynthesis of muscle protein was not clear-cut related to circulating GH, blood IGF-I and locally produced IGF-I mRNA [9]. Others have however reported that a one hour iv. infusion of IGF-I to overnight starved mice increased muscle protein synthesis to levels comparable to fed mice [32]. Furthermore, reduced free plasma IGF-I by 50% of normal levels, by infusion of IGF binding protein 1, decreased muscle protein synthesis by 25% with decreased phosphorylation of p70s6 kinase [33], while provision of IGF-I/IGFBP-3 complex improved sepsis induced muscle catabolism [34]. However, plasma IGF-I did not diverge from basal levels in humans who received a drink of essential amino acids at a time when muscle protein synthesis was stimulated by more than 100% [35]. Another study in humans evaluated effects by IGF-I when infused directly into arm muscle bed compared to systemically raised IGF-I [36], where protein synthesis was only increased subsequently to locally infused IGF-I. A study, based on microdialysis, revealed that concentrations of free IGF-I in muscle interstitial fluid were 20 fold higher than simultaneous plasma levels, supporting that circulating IGF-I is not directly determining rates of protein synthesis during feeding [37]; observations in line with our own findings that infused amino acids improved protein balance across human muscles independently of plasma insulin, IGF-I and IGF-I-binding proteins-1 and −3 [3].

A compensatory factor in the present study may be that increased levels of growth hormone (GH) in liver-IGF-I knockouts, observed in the present and a previous study, would counteract subnormal IGF-I levels in the circulation [15] since cell experiments indicate that GH phosphorylates similar or the same downstream targets for protein synthesis as amino acids and IGF-I through PI3-kinase dependent transduction [38]. Also, GH treatment increased fractional muscle protein synthesis in fed but not in starved pigs [39]. However, in a previous study we used mice where circulating levels of both GH and IGF-I were low and found that fractional muscle protein synthesis increased normally to expected levels after food intake [9]. Re-synthesis of muscle protein following starvation-refeeding did not relate to plasma GH concentrations in either knockouts or wild type mice. Therefore, we find it unlikely that GH explains rapid alterations in the feeding related muscle protein synthesis evaluated in the present model.

We found no difference between liver IGF-I deficient and wild type mice regarding translational control of muscle protein synthesis. Both liver IGF-I knockouts and wild type mice showed increased phosphorylation of 4EBP1 protein and decreased association of the 4E-BP1 · eIF4E complex. Also, p70s6 kinase phosphorylation was increased similarly in both groups including upstream phosphorylation of mTOR^ser2448^. Thus, effects by circulating IGF-I were less critical for the control of translation initiation compared to other possible factors such as plasma or extracellular amino acids, although consistent changes in plasma amino acids were not observed among IGF-I deficient and wild type mice at starvation-refeeding. However, we did not measure eIF4G · eIF4E complexes, which may be influenced by IGF-I as reported by others using a hindlimb perfusion model, where physiologic concentrations of IGF-I (10 nM) in the presence of amino acids were unable to dissociate 4E-BP1 from eIF4E, but increased the assembly of eIF4G-4E complex and stimulated protein synthesis [40].

Both liver IGF-I knockouts and wild type mice showed increased muscle expression of the IGF-IR during starvation, which usually reflects cellular processes related to regeneration and hypertrophy of muscle cells [6]. This increased receptor expression may reflect a positive feedback control of IGF-I signaling secondary to decreased IGF-I protein close to membranes; a conclusion supported by observations in cultured cells on increased IGF-IR number and promoted amino acid transport related to decreased extracellular concentration of amino acids, particularly glutamine [30,41]. Increased transcript and IGF-IR protein levels have also been reported in response to exercise [42-44], but information of similar changes in relation to feeding are sparse, although equivalent observations have been reported [45,46]. Thus, IGF-IR expression appears more tightly controlled than tissue IGF-I expression in skeletal muscle metabolism in response to starvation – refeeding. By contrast to feeding, a recent report in mice with non-functional IGF-IR showed that load-induced hypertrophy occurred [47], which suggested that increased protein synthesis following exercise was not entirely dependent on IGF-IR signaling.

There were no changes in either PI3K or AKT expression during starvation-refeeding, while mTOR transcript expression was up-regulated in liver-IGF-I knockouts during starvation, but without apparent change in protein phosphorylation. This up-regulation in starved knockouts may therefore reflect compensatory effects unrelated to phosphorylation of mTOR, secondary to consistently decreased circulating IGF-I. If so, it remains to be explored which factor(s) that up-regulates mTOR expression in muscle tissue when endocrine IGF-I is declined, particularly during starvation of knockout mice (Figure 2C).

## Conclusion

In conclusion, our present results confirm previous reports that alteration in muscle protein synthesis during starvation–refeeding relates to alteration in muscle mTOR signaling, but not to Akt and PI3-kinase. Present findings also support our previous conclusions that muscle produced IGF-I/IGF-IR are related to translation initiation of muscle proteins at oral feeding. [5]. New information is that endocrine IGF-I is not, critical for this effect. It remains to determine to what extent local muscle IGF-I signaling is critical, since systemically IGF-I ^(+/−)^ knockout mice had normal fractional synthesis rate (%/hrs) in skeletal muscles at refeeding [9]. Whether a lack of effects by liver derived IGF-I may be overcome by alternative signals upstream to mTOR remains also to be determined, but insulin and GH are probably not such candidates.

## Abbreviations

AEBSF: 4-(2-aminoethyl)benzenesulfonyl fluoride; EGTA: Ethylene-bis[oxyethylenenitrilo]tetraacetic acid; KCl: Potassium chloride; NaF: Sodium fluoride; eIF4E: Eukaryotic initiation factor 4E; eIF4G: Eukaryotic initiation factor 4G; 4E-BP1: Eukaryotic initiation factor 4E binding protein 1; p70s6k: Ribosomal protein S6 Kinase, 70-kDa; Li-IGF-I: Liver derived IGF-I; IGF-IR: IGF-I receptor; PiPc: Polyinosinic- polycytidylic acid; mTOR: mammalian target of Rapamycin; PI3K: Phosphatidylinositol 3-kinase; Akt: Proteinkinase B-alpha; DTT: Ditiotreitiol; EDTA: Ethylenediaminetetraacetic acid.

## Competing interests

The authors’ declare that they have no competing interests.

## Authors’ contributions

B.I carried out the analyses, calculated statistics, and drafted manuscript, JS CO generated the transgenic mice KL. Conceived of the study and drafted manuscript. All authors participated in study design and critical revision of manuscript. All authors have read and approved the manuscript.
